# Tuning macrophages for atherosclerosis treatment

**DOI:** 10.1093/rb/rbac103

**Published:** 2022-12-13

**Authors:** Fei Fang, Crystal Xiao, Chunli Li, Xiaoheng Liu, Song Li

**Affiliations:** Institute of Biomedical Engineering, West China School of Basic Medical Sciences & Forensic Medicine, Sichuan University, Chengdu 610041, China; Department of Bioengineering, University of California, Los Angeles, Los Angeles, California 90095, USA; Department of Medicine, University of California, Los Angeles, Los Angeles, California 90095, USA; Department of Bioengineering, University of California, Los Angeles, Los Angeles, California 90095, USA; Department of Medicine, University of California, Los Angeles, Los Angeles, California 90095, USA; Institute of Biomedical Engineering, West China School of Basic Medical Sciences & Forensic Medicine, Sichuan University, Chengdu 610041, China; Institute of Biomedical Engineering, West China School of Basic Medical Sciences & Forensic Medicine, Sichuan University, Chengdu 610041, China; Department of Bioengineering, University of California, Los Angeles, Los Angeles, California 90095, USA; Department of Medicine, University of California, Los Angeles, Los Angeles, California 90095, USA

**Keywords:** macrophages, atherosclerosis, drug delivery system, immune engineering

## Abstract

Atherosclerosis is a chronic inflammatory vascular disease and a leading cause of death worldwide. Macrophages play an important role in inflammatory responses, cell–cell communications, plaque growth and plaque rupture in atherosclerotic lesions. Here, we review the sources, functions and complex phenotypes of macrophages in the progression of atherosclerosis, and discuss the recent approaches in modulating macrophage phenotype and autophagy for atherosclerosis treatment. We then focus on the drug delivery strategies that target macrophages or use macrophage membrane-coated particles to deliver therapeutics to the lesion sites. These biomaterial-based approaches that target, modulate or engineer macrophages have broad applications for disease therapies and tissue regeneration.

## Graphical abstract

**Figure:**
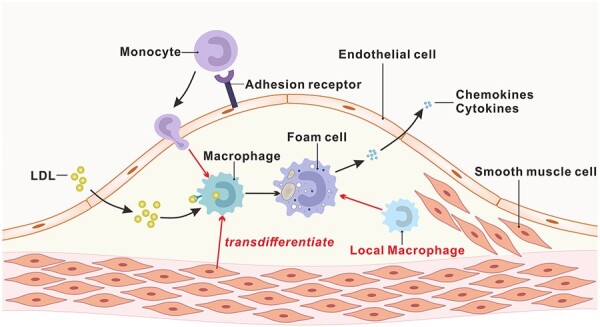

## Introduction

Atherosclerosis is a chronic inflammatory disease in arteries [1], characterized by intimal thickening, smooth muscle cell (SMC) proliferation, lipid accumulation and plaque formation. It mainly occurs in areas with the disturbed non-laminar flow and endothelial cell damage [2]. Most immune cells are believed to be involved in the occurrence and development of atherosclerosis, including monocytes, macrophages, dendritic cells, T cells and B cells [3]. Macrophages play an essential role in the development of atherosclerosis and are the dominant immune cell type within the atherosclerotic plaque [4]. Macrophages display distinct functional phenotypes, the classic pro-inflammatory M1 macrophages being localized near the lipid core, while anti-inflammatory M2 macrophages are more enriched in neo-angiogenic areas [5]. There is evidence that the macrophage phenotype may modulate the remodeling of the atherosclerotic lesion [6]. Therefore, targeting macrophages, tuning macrophage phenotype and engineering macrophage or its components as a drug carrier are promising approaches to treat atherosclerosis. Here, we review macrophage phenotype and functions in atherosclerotic lesions and discuss therapeutic approaches in engineering macrophages and drug delivery.

## Macrophages in atherosclerosis

### Source of macrophages in atherosclerosis

**Figure 1.**
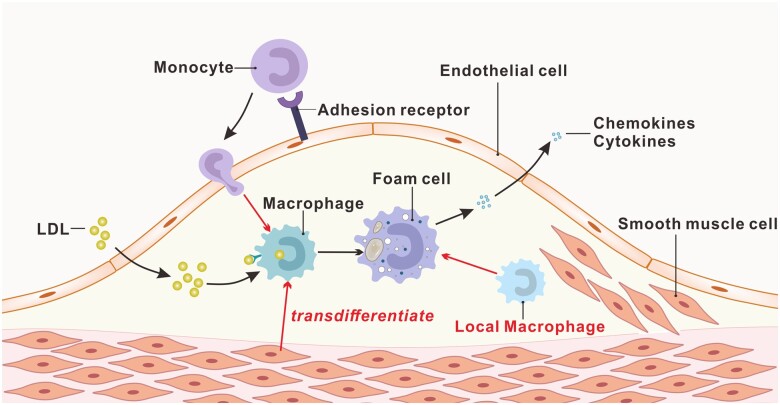
Schematic diagram of the source of macrophages in atherosclerosis.

Macrophages are the predominant immune cell type at atherosclerotic plaques and play a central role in the progression of atherosclerotic inflammation. The sources of macrophages at the plaque are mainly divided into three categories: (i) the monocytes in the circulating blood; (ii) vascular SMCs transdifferentiation; and (iii) the artery-resident macrophages (Fig. 1).

The most important source of macrophages in atherosclerosis is monocytes in circulating blood. Monocytes can be recruited by activated endothelial cells and differentiate into macrophages in the presence of macrophage colony-stimulating factor (M-CSF) [7, 8]. Because the plaque macrophage content is directly correlated with the number of monocytes, enhanced monocytosis causally accelerates the progression of atherosclerosis [9–12]. Medullary hematopoiesis has long been considered the primary source of monocytes in atherosclerosis, and hyperlipidemia increases monocyte number in circulation by activating hematopoietic stem and progenitor cell proliferation [12–14]. However, the extramedullary hematopoietic function has also been shown to promote atherosclerosis due to the increase of monocytes derived from bone marrow and maturity in the spleen [15]. Approximately 30% of the monocytes aggregate in the aorta, and 45 ± 15% of the monocytes found in the blood are derived from the spleen after 24 hours of allogeneic spleen transplantation in mice. Therefore, the sources of monocyte-derived macrophages may include medullary hematopoiesis and extramedullary hematopoietic production.

Vascular SMCs are a primary cell type that presents in all stages of atherosclerosis plaque. As early as 2003, it was demonstrated that *in vitro* mouse SMCs loaded with cholesterol could transdifferentiate into macrophage-like cells [16]. After 72 h of cholesterol treatment, the expression of SMC markers α-actin, α-tropomyosin, myosin heavy chain and calponin H1 were downregulated, and macrophage-like markers such as CD68 and Mac-2 were upregulated. In addition, studies have shown that more than 50% of foam cells in human atherosclerotic plaque are derived from SMCs [17]. These SMCs have lower ATP-binding cassette transporter A1 (ABCA1) expression and weaker cholesterol transport capacity than myeloid lineage cells [17]. A recent study also shows that the contribution of SMCs to total foam cells in ApoE^−/−^ mice is similar to leukocyte-derived foam cells by using SMC-lineage tracing [18]. Although the involvement of SMC-derived macrophage-like cells in atherosclerosis has been established, the mechanisms and the contributions of these cells to disease progression have yet to be elucidated.

In addition, artery-resident macrophages are another source of atherosclerotic macrophages. The artery macrophages arise embryonically from C-X3-C motif chemokine receptor 1-positive (CX3CR1+) precursors derived from bone marrow monocytes, which migrate to the blood vessel wall and settle shortly after birth [19]. Although the specific role of these resident macrophages in the progression of atherosclerosis is not yet clear, it is speculated that some of these macrophages could uptake lipids and be converted into foam cells [20].

### The functions of macrophages in atherosclerosis

The macrophages in atherosclerosis plaque have different functions due to their origin and diverse phenotype. The oxidized low-density lipoprotein (oxLDL) plays a crucial role in atherosclerosis by interacting with endothelial cells, macrophages and SMCs [21]. In the early stages of atherosclerosis, the major function of macrophages is to internalize and degrade the sub-endothelially retained lipoproteins. Macrophages could engulf oxidized low-density lipoproteins through scavenger receptors such as CD36 (also known as fatty acid translocase), SR-A1 (scavenger receptor A1, also known as MSR1), scavenger receptor B1 (SR-B1), LDL receptor-related protein 1 (LRP-1) and lectin-like oxLDL receptor-1 (LOX-1) [22]. The internalized oxLDL is hydrolyzed into cholesterol and free fatty acids by lysosomal acid lipase. Some free cholesterol is catalyzed by cholesterol acyltransferase-1 (ACAT-1) and stored in the endoplasmic reticulum of macrophages as lipid droplets. The remainder, free cholesterol, can be effluxed by cholesterol ATP-binding cassette (ABC) transporters ABCA1, ABCG1 and SR-B1 [23]. However, under pathological conditions such as high blood pressure, smoking, diabetes and high oxLDL, the macrophage metabolism becomes dysfunctional, causing a large amount of cholesteryl ester to accumulate in the macrophages, eventually leading to the formation of foam cells [24].

Inflammatory macrophages can promote the progression of atherosclerosis by expressing surface markers, including primary histocompatibility complex class II, Fc receptor CD64 and costimulatory molecules CD80 and CD86, and secrete chemokines and cytokines to promote vascular inflammation, including monocyte chemoattractant protein-1 (MCP-1), interleukin-1 (IL-1), IL-6, IL-12, IL-15, IL-18, IL-23 and tumor necrosis factor α (TNF-α) [4, 25]. These surface molecules can act as immune recognition receptors to activate the acquired immune response pathway, amplifying the local inflammatory response [26, 27]. Macrophages in atherosclerosis can be activated by oxLDL via toll-like receptors and the nuclear translocation of NF-κB [28]. oxLDL is recognized by CD14–TLR4–MD2 and the interaction triggers cytoskeletal rearrangement and the production of TNF-α, IL-6 and IL-10 [29]. In addition to oxLDL, cholesterol crystals could activate NACHT, LRR and PYD domain-containing protein 3 (NLRP3) inflammasome in foam cells, leading to the release of IL-1β [30, 31]. Therefore, regulating the response of macrophages to lipids and other pro-inflammatory cytokines can be a potential target for atherosclerosis treatment.

Macrophage apoptosis is critical in forming the necrotic core, an essential feature of vulnerable lesion plaque. The causes of death of macrophages in atherosclerosis include oxidative stress, high concentration of cytokines, oxidized low-density lipoprotein, Fas ligand-induced apoptosis and endoplasmic reticulum stress [32]. Endoplasmic reticulum stress is strongly correlated with macrophages’ death and atherosclerotic nuclei formation through activating UPR (unfolded protein response), resulting in the activation of CAAT/enhancer-binding protein (C/EBP) homologous protein [32, 33]. In addition, macrophages are involved in forming vulnerable plaques, which can eventually lead to the rupture of atherosclerotic plaques. The matrix metalloproteinases (MMPs) secreted by macrophages are related to the thinning of the fiber cap and the formation of vulnerable plaque [34]. Macrophages and activated MMPs (MMP-2 and MMP-9) are found in the shoulder area of atherosclerotic instability plaques [35]. MMP-9 participates in different stages of atherosclerosis. For example, macrophages-derived MMP-9 promotes the infiltration of macrophages into the lesion, thereby promoting the progression in the early stage of atherosclerosis [36]. MMP-1, MMP-3, MMP-8, MMP-13 and MMP-14 have also been confirm to contribute to plaque instability, although their effects are somewhat different [37]. The hydrolysis of extracellular matrix (ECM) by MMPs is considered a reasonable assumption for the instability and rupture of atherosclerotic plaques. Moreover, other proteases such as cathepsin K are also involved in atherosclerosis development. Cathepsin K can induce atherosclerosis by regulating the re-distribution of ECM, and its expression is regulated by the disturbed flow-mediated integrin-cytoskeleton-NF-κB signaling axis [38].

Macrophages play many essential roles in the innate and adaptive immune response. Macrophages can serve as antigen-presenting cells to present major histocompatibility complex class I (MHC-I) molecules to naive CD8^+^ T cells and MHC-II molecules to naive CD4^+^ T cells [3]. CD8^+^ killer T cells induce apoptosis and necrosis of target cells through cytotoxins or cytokines, thereby exacerbating the inflammatory response in atherosclerotic plaques and driving lesion progression and instability [39, 40]. CD4^+^ T cell subsets can differentially affect the progression of atherosclerosis through immune activation or immunosuppression or by helping B cells produce antibodies [40].

**Figure 2.**
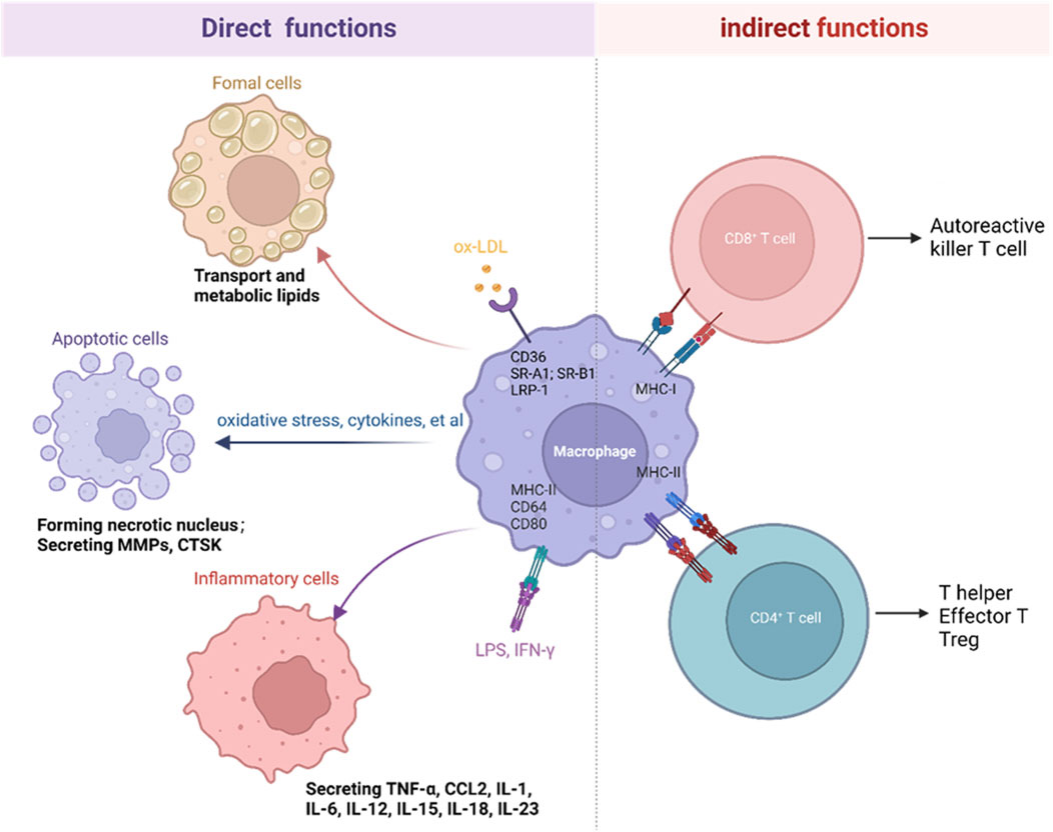
The functions of macrophages in atherosclerosis (created with biorender.com).

In short, macrophages play an essential role in lipid metabolism, inflammatory responses, necrotic core formation and plaque instability during the development of atherosclerosis (Fig. 2).

### The phenotype of macrophages in atherosclerosis

**Figure 3.**
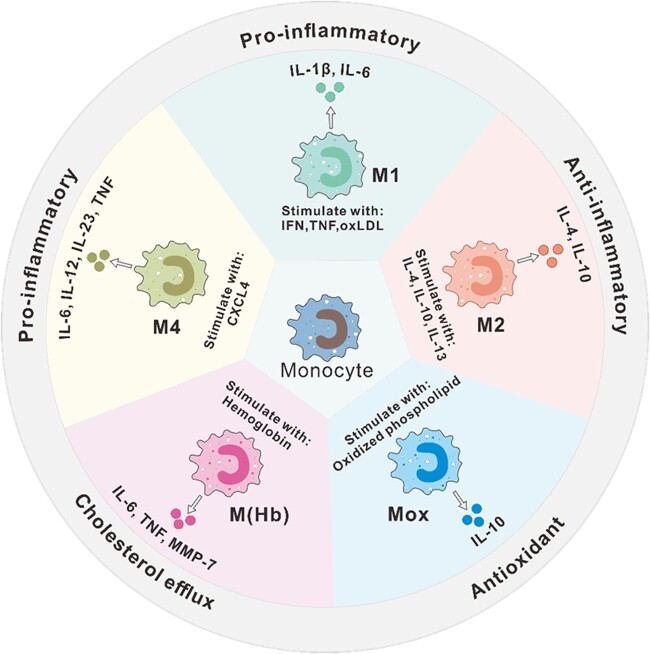
Major macrophage subtypes in atherosclerotic lesions. M1, M1 macrophages; M2, M2 macrophages; Mox, Mox macrophages; M(Hb), hemoglobin (Hb)-stimulated macrophages; M4, M4 macrophages.

Macrophages in the atherosclerosis plaque are highly heterogeneous and plastic. Many subtypes of macrophages in atherosclerosis are classified according to surface markers and secreted proteins (Fig. 3). Classically activated M1-type macrophages are induced by Th1 cytokines such as interferon (IFN)-γ and TNF-α. M1 macrophages, also known as inflammatory macrophages, promote sustained inflammation by secreting pro-inflammatory cytokines, such as IL-6, IL-1β, TNF-19, IL-12 and IL-23 [41]. Surface markers of M1 macrophages include CD80, CD86, CD64, CD16 and CD32. M2-type macrophages as anti-inflammatory macrophages could counteract the inflammatory response produced by M1 macrophages, which are involved in tissue repair, phagocytosis, angiogenesis and fibrosis promotion [5]. Surface markers of M2 macrophages include CD163 and CD206. M2 macrophages were initially thought to be mainly induced by IL-4 and IL-13 produced by Th2 cells. Subsequent research found that it can be further divided into three subtypes [42, 43], including M2a, M2b and M2c: M2a macrophages are induced by the activation of the STAT6 pathway by IL-4 and IL-13 through the co-receptor IL-4Rα and are mainly involved in tissue repair and antifungal responses; M2b macrophage subtypes are induced by immune complexes with IL-1β or lipopolysaccharide and are mainly involved in regulating immune responses; and M2c macrophages are induced by IL-10 and have strong anti-inflammatory and phagocytic effects. A common feature of all M2 macrophages is the low production of IL-12 and high production of IL-10 and transforming growth factor β (TGF-β). However, M2b macrophages also produce high levels of pro-inflammatory cytokines, including IL-1, IL-6 and TNF [44].

In addition to M1 and M2 macrophages, other subtypes of macrophages are also found in atherosclerosis. For example, a new macrophage phenotype named Mox is triggered by the activation of NFE2L2. Compared with M1 and M2, Mox shows a different gene expression pattern and reduced phagocytosis and chemotaxis [45]. Mox macrophages account for ∼30% of the total number of macrophages in advanced atherosclerotic lesions in mice.

There is evidence that the macrophage phenotype is affected by the microenvironment in the atherosclerotic plaque. The area of atherosclerotic plaque is rich in lipids and their derivatives, which can regulate macrophage plasticity. Cholesterol crystals can induce polarization of M1 macrophages, activate NLRP3 inflammasomes to promote inflammation, and release IL-1 and IL-8 [30]. The increased accumulation of oxLDL induces a transformation from M2 macrophages to M1 phenotype and increases the secretion of pro-inflammatory cytokines IL-6, IL-8 and MCP-1 [46, 47]. Cholesterol linoleic acid, the primary cholesterol ester in atherosclerotic plaques, activates M1 polarization via a MAP kinase-dependent mechanism, and its oxidation products (7-ketocholesteryl-9-carboxynonanoate) can promote an anti-inflammatory phenotype by inducing the expression and secretion of TGF β1 [48, 49]. In addition, a new macrophage phenotype, referred to as M(Hb) (which is induced by hemoglobin and expresses high levels of mannose receptor-1 CD163 and scavenger receptor cysteine-rich type 1 protein M130 (CD163)), was found in the bleeding area of human atherosclerotic plaques [50]. M(Hb) macrophages downregulate the expression of proteins related to lipid uptake, such as scavenger receptors, and have a strong cholesterol efflux ability, thus reducing the production of foam cells [50]. Furthermore, by reducing the intracellular iron content, M(Hb) can reduce reactive oxygen species (ROS) production, thereby regulating the conversion of macrophages to a non-foam cell phenotype to enhance an anti-atherosclerotic effect [50, 51]. The growth factors in the atherosclerotic plaque are essential regulators of macrophage differentiation and polarization. M-CSF dominates the early lesions and induces an M2-like phenotype, while the increase in granulocyte M-CSF expression during the development of atherosclerosis tends to induce an M1-like phenotype [5]. In addition, some chemokines also regulate the phenotype of macrophages. Platelet-derived chemokine CXCL4 induces a new type of human macrophage phenotype, M4 macrophage, with the expression of surface markers such as S100A8, CD206 and MMP-7. M4 macrophage irreversibly loses the expression of CD163 protein and shows the characteristics of promoting atherosclerosis and has been associated with plaque instability [52, 53].

## Tuning macrophages for atherosclerosis therapy

### Modulation of macrophage phenotypes

The dynamic balance of M1/M2 macrophages has an important influence on the progression of atherosclerosis. Histological analysis of human plaques revealed that M1 macrophages are rich in lipids and are located in a different area from M2 macrophages [54]. M1 and M2 macrophage polarized studies have simplified the view that M1 macrophages promote inflammation and M2 macrophages reduce inflammation [55]. It is generally accepted that atherosclerosis can be prevented by promoting the M2 polarization of macrophages. Therefore, in addition to the aforementioned cytokines (Fig. 3), chemical compounds, proteins, microRNAs and exosomes have been explored for atherosclerosis treatment (Table 1).

**Table 1.** Regulating macrophage polarity to treat atherosclerosis

Drug	Polarization	Pathway	Model
Crocin	M1↓ M2↑	AMPK	Rat [56]
Ginsenoside Rb1	M2↑	STAT	ApoE^−/−^ mice [57]
Ginsenoside Rg3	M1↓ M2↑		ApoE^−/−^ mice [58]
Geniposide	M1↓ M2↑	FOX-MAPK	New Zealand rabbits [59]
Protocatechuic acid	M1↓ M2↑	PI3K-Akt-NF-κB	ApoE^−/−^ mice [60]
5-Amino abietic acid	M1→M2	ROS-AMPK-mTORC1	ApoE^−/−^ mice [61]
Kallistain	M1↓ M2↑	KLF4	ApoE^−/−^ mice [62]
Thioredoxin	M2↑		ApoE^−/−^ mice [63]
Melatonin	M1↓	AMPKα-STATs	ApoE^−/−^ mice [64]
miR-33	M2↑	AMPK	Hypercholesterolemic mice [66]
MSCs-derived exosomes	M1↓ M2↑	miR-let7-HMGA2-NF-κB	ApoE^−/−^ mice [70]
MSCs-derived exosomes	M2↑	miR-21a-5p-KLF6-ERK1/2	ApoE^−/−^ mice [71]

Recent studies showed that chemical compounds such as plant extracts can regulate the polarizing phenotype of macrophages. In a rat model of coronary atherosclerosis induced by vitamin D3, crocin can reduce the number of M1 macrophages and increase the polarization of M2 macrophages, thus minimizing inflammation and alleviating atherosclerosis [56]. *In vitro*, Ginsenoside Rb1 treatment increase the expression of two classic M2 macrophage markers, arginase 1 (Arg-1) and macrophage mannose receptor (CD206), while the expression of iNOS (M1 macrophages) is reduced [57]. *In vivo*, the administration of Rb1 promotes the stability of atherosclerotic lesions and increases the M2 macrophage phenotype population [57]. Another active component in ginseng is Rg3, which has been confirmed to improve the stability of atherosclerosis lesions and reduce plaque burden, accompanied by an increase in M2 macrophages and a decrease in M1 macrophages [58]. In addition, some other natural active substances such as geniposide [59], protocatechuic acid [60] and 5-amino abietic acid [61] have also been found to induce the polarization of intraplaque macrophages to M2 type to inhibit atherosclerosis.

Proteins have also been shown to regulate the polarity of macrophages for the treatment of atherosclerosis. For example, the adenovirus vector containing the human kallistatin gene can be delivered into atherosclerotic ApoE^−/−^ mice through the tail vein injection; the high expression of kallistatin protein upregulates the expression of M2 macrophage markers such as IL-10 and Arg-1, and downregulates the expression of M1 macrophage markers such as iNOS and MCP-1 [62]. Thioredoxin (Trx) is an oxidative stress-limiting protein with anti-inflammatory and anti-apoptotic properties. Injection of Trx-1 into ApoE^−/−^ mice causes the phenotype change of macrophages into M2 instead of M1 in the diseased area and significantly reduces the aortic lesion area [63]. Another study demonstrates that melatonin (an indoleamine hormone secreted by the pineal gland) could alleviate inflammation in atherosclerotic plaques by inhibiting the differentiation of macrophages in the plaque to the pro-inflammatory M1 phenotype [64].

The inhibition of miR-33 can induce autophagy in macrophages [65], and induce M2 polarization, which is mediated by energy sensor AMP-activated protein kinase (AMPK) [66]. The treatment of hypercholesterolemia mice with miR-33 inhibitors for eight weeks results in the accumulation of inflammation-suppressing M2 macrophages and FOXP3 Tregs in plaque areas and inhibits the progression of atherosclerosis [66]. This study suggests that miR-33 inhibitors have the potential for atherosclerosis treatment.^+^

Recently, studies have shown that exosomes can regulate the phenotypic transformation of macrophages and affect disease progression [67, 68]. The adipose tissue-derived exosomes induce RAW264.7 macrophages to switch to the M1 phenotype, increase the secretion of pro-inflammatory cytokines (TNF-α, IL-6), and exacerbate atherosclerosis in hyperlipidemic ApoE^−/−^ mice [69]. In contrast, mesenchymal stem cell-derived exosomes can induce more M2-type macrophage markers, reduce the infiltration of M1 macrophages and shrink the area of atherosclerotic plaques in ApoE^−/−^ mice [70]. A similar study finds that mesenchymal stem cell-derived exosomes containing miR-21a-5p can promote macrophage polarization and reduce macrophage infiltration by targeting KLF6 and ERK1/2 signaling pathways, thereby attenuating the development of atherosclerosis [71].

### Induction of macrophage autophagy

Autophagy is a process of self-digestion by cells to maintain cell homeostasis and degrade mistranslated proteins and organelles in cells. Autophagy is involved in clearing cholesterol deposits in blood vessel tissues at the early stage of atherosclerosis [72]. The destruction of essential autophagy-related genes (Atg5, Atg7, Atg14) in macrophages accelerates the formation of atherosclerotic plaques in mice, and the macrophage-specific Atg5-knockout or Atg14-knockout mice show increased plaques apoptosis and larger necrotic cores [73–75]. Macrophage autophagy can be used as a potential strategy for treating atherosclerosis, as it plays an essential protective role in atherosclerosis progression [76] (Table 2).

**Table 2.** Induction of macrophage autophagy for atherosclerosis treatment

	Function	Pathway	Model
Trehalose	Induces autophagy lysosomal biogenesis	TFEB-P62/autophage	ApoE^−/−^ mice [79]
Hypericin	Induces autophagy and decreases ROS produce	AMPK-mTOR/AKT-mTOR	THP-1 cell [80]
Cordycepin	Induces autophagy and promot cholesterol efflux	AMPK-mTOR	ApoE^−/−^ mice [81]
Ursolic acid	Suppresses IL-1β secretion; promotes cholesterol efflux	Atg5/Atg16l1-autophagy	LDLr^−/−^ mice [82]
Ginsenoside Rb1	Reduces lipid accumulation	MAPK/Atg5-autophagy	ApoE^−/−^ mice [83]
Isorhamnetin	Enhances the lysosomal function		J774.1 cell [84]
Rosuvastatin	Inhibit lipid accumulation and polarization	PI3K-Akt-mTOR	ApoE^−/−^ mice [92]
Hydroxy safflower yellow A	Induce autophagy and inhibit inflammation	PI3K-Akt-mTOR	THP-1 cell [93]
Resveratrol	Improve efferocytosis of apoptotic RAW264.7 cells	Sirt1-autophagy	RAW264.7 cell [95]
Berberine	Inhibit macrophages apoptosis	Sirt1/TFEB-autophagy	Peritoneal macrophages [96, 97]
miR-33	Induces cholesterol efflux	Atg5/TFEB-autophagy	LDLr^−/−^ mice [65]
Arsenic trioxide	Induces autophagy and decreases plaque lesion	PI3K-AKT-mTOR	ApoE^−/−^ mice [101]

**Table 3.** The targeted delivery system for atherosclerosis

Target	Ligand	Carrier	Drug	Model
Mannose receptor	Mannose	Dendrimeric NPs	LXR agonist, T0901317	LDLr^−/−^ mice [104]
CD36 receptor	PtdSer	rHDL	SR-A siRNA/Pitavastatin	ApoE^−/−^ mice [108]
CD36 receptor	CD36 antibody	mPEG-PAsp	Anti-PAK1 siRNA	ApoE^−/−^ mice [109]
CD44 receptor	Hyaluronic acid	Hyaluronic acid	LOX-1 siRNA	ApoE^−/−^ mice [112]
CD44 receptor	Hyaluronic acid	Hyaluronic acid	Atorvastatin	ApoE^−/−^ mice [113]
CD44 receptor	Hyaluronic acid	PtBA100-b-PEHA60	Simvastatin	ApoE^−/−^ mice [114]
Stabilin-2	S2P peptide (CRTLTVRKC)	DSPE-PEG, PLGA	CaMKIIγ siRNA	LDLr^−/−^ mice [116]
Folate receptor (macrophage)	Folate	PAMAM-PEG		ApoE^−/−^ mice [117]
Macrophage	CGNKRTRGC	Simian virus 40	Hirulog	ApoE^−/−^ mice [118]
Monocytes and macrophages LFA-1	Anti-CD11a antibody	ADC	LXR agonist	in vitro macrophages [119]
Integrin αvβ3	cRGDfK peptide	PLGA-PEG	Rapamycin	ApoE^−/−^ mice [120]
Scavenger receptor	PtdSer	Latex NPs	Rosiglitazone/ tamoxifen / paclitaxel	ApoE^−/−^ mice [121]
CD36 receptor	Phosphatidylcholine	Phosphatidylcholine	Epigallocatechin gallate	LDLr^−/−^ mice [122]
SR-B1	Apolipoprotein A-I	Phospholipid	Anti-miR155	*in vitro * macrophages [123]

Overexpression of a transcription factor EB (TFEB) that drives autophagy-lysosomal biogenesis in macrophages can reduce arteries atherosclerosis [77], help reduce lipid-mediated lysosomal dysfunction, increase cholesterol efflux and inactivate inflammasome activation [78]. Recent studies have shown that trehalose can be used as an autophagy inducer to promote the nuclear translocation of TFEB and atheroprotective properties [77, 79], although the precise mechanism by which this disaccharide induces transcriptional activation of TFEB is unclear. In addition, hypericin [80], cordycepin [81], ursolic acid [82], ginsenoside Rb1 [83] and isorhamnetin [84] have been shown to regulate macrophage autophagy leading to anti-atherosclerotic effects.

The mTOR is a highly conserved serine/threonine-protein kinase belonging to the phosphoinositide kinase-related kinase (PIKK) family and the core of autophagy regulation [85, 86]. Knockout of mTOR by siRNA will activate autophagy-related proteins, downregulate gene expression of MMP-2 and MCP-1, promote macrophage autophagy and stabilize atherosclerotic plaques [87]. One of the mTOR inhibitors, rapamycin, is one of the most in-depth and well-known autophagy inducers. Rapamycin or rapalogs stimulate autophagy through the inhibition of mTOR. Delivery of rapamycin via a blood vessel-eluting stent may lead to the selective induction of macrophage autophagy and a significant reduction of macrophages without changing the content of SMCs in atherosclerotic plaques [88]. Everolimus (a rapalog of rapamycin) eluting stents have been shown to prevent vascular restenosis after heart transplantation or other heart surgery [89–91]. In addition, rosuvastatin can induce autophagy to improve the conversion of macrophages to foam cells by inhibiting PI3K/Akt/mTOR signaling pathways [92]. Knockout of mTOR by siRNA will activate autophagy-related proteins, downregulate genes including MMP-2, MCP-1 and tissue factor, promote macrophage autophagy and stabilizes atherosclerotic plaques [87].

Some natural plant extracts have been shown to induce autophagy to protect against atherosclerosis. For example, hydroxy safflower yellow A induces autophagy through PI3K/Akt/mTOR signaling pathway and inhibits the inflammatory response of macrophages [93]. After hydroxy safflower yellow A treats THP-1 macrophages, autophagy is induced, as shown by the transformation of LC3-II/LC3-I, the increased expression of beclin 1, the degradation of p62, the formation of autophagic vesicles and the lower expression of inflammatory factors [93]. Resveratrol is another essential autophagy inducer, which acts through the mTOR signaling pathway, and plays a vital role in anti-atherosclerosis and vasodilation by inducing autophagy [94]. Resveratrol induces autophagy by activating *sirt1*, improving the oxLDL uptake and enhancing efferocytosis of apoptotic RAW264.7 cells [95], suggesting that *sirt1* could be used as a novel therapeutic target for atherosclerosis treatment [95]. Recent studies have also shown that the anti-atherosclerotic effect of berberine may be through the activation of the Sirt1-TFEB or AMPK/mTOR signaling pathway to induce autophagy and inhibit the inflammatory response of macrophages [96, 97]. In addition to inducing autophagy, resveratrol could inhibit the inflammation of Ana-1 murine macrophages caused by cholesterol, inhibit the expression of IL-1β in macrophages and enhance the reverse cholesterol transport of macrophages [98]. An *in vivo* study also shows that ApoE^−/−^ mice with 24 weeks of oral resveratrol eliminate intestinal fatty acids and monoglycerides accumulation in atherosclerotic lesions [99]. The possible molecular mechanism is that resveratrol activates the peroxisome proliferator-activated receptors signal to enhance the cholesterol efflux mediated by ABC transporter A1/G1, thereby reducing the accumulation of total cholesterol, esterified cholesterol, and neutral lipids triggered by oleic acid in mouse RAW264.7 macrophages [99]. It is worth noting that inhibiting mTOR signaling may also cause other side effects due to the complexity of the mTOR signaling.

MicroRNAs inhibit autophagy by transcriptionally inhibiting autophagy-related upstream genes [65, 100]. For example, miR-33 can inhibit the autophagy of macrophages in atherosclerosis. Therefore, treating atherosclerotic LDLr^−/−^ mice with anti-miR-33 can restore autophagy defects in plaque macrophages, increase lysosome biogenesis and reduce plaque necrosis through autophagy-dependent mechanisms [65].

In addition, some classic drugs are also used to treat atherosclerosis. A typical example is that arsenic trioxide has been proven to induce macrophage autophagy and atherosclerotic protection by regulating ROS-dependent nuclear translocation of TFEB and the AKT/mTOR pathway [101]. Furthermore, the arsenic trioxide drug-eluting stents could alleviate in-stent restenosis by selectively inhibiting the proliferation of SMCs [102].

## Targeting macrophages for drug delivery

**Figure 4.**
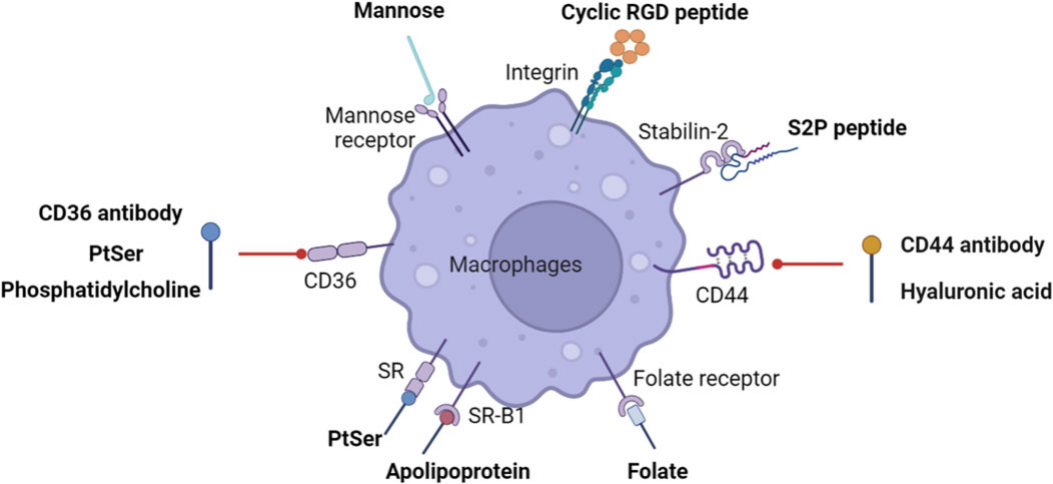
Cell surface molecules for atherosclerotic macrophage targeting (created with biorender.com).

Macrophages are major cell types in atherosclerotic plaque, so targeting macrophages is a potential strategy for treating atherosclerotic lesions locally (Table 3). Macrophages express various protein receptors, some of which can be used as potential therapeutic targets, such as the mannose receptor, macrophage scavenger receptor (SR), CD44 receptor and folate receptor [103] (Fig. 4). Mannose receptor is a C-type lectin that exists on the surface of macrophages. In atherosclerotic plaques, mannose-functionalized nanoparticles can be specifically uptaken by macrophages [104]. The targeted delivery of liver-x-receptor (LXR) agonist T0901317 significantly reduces the mouse aortic arch’s plaque area and the expression of MMP-9 in the plaque [104]. This LXR agonist T0901317 inhibits atherosclerosis, but off-targeting effects can induce liver steatosis and hypertriglyceridemia [105]. Therefore, a novel platform named D-Nap-GFFY encapsulating T0901317 nanofiber hydrogel is developed to target macrophages in atherosclerosis sites and reduce lesions without effect on hepatic lipogenesis [106].

Class B scavenger receptor CD36 and scavenger receptor A (SR-A) are mainly responsible for the uptake of modified LDL by macrophages in atherosclerosis [107]. Phosphatidylserine (PtdSer) can specifically target the CD36 receptor of macrophages. A dual-functional nano-platform uses phosphatidylserine to modify Rhdl (recombinant high-density lipoproteins) and is loaded with pitavastatin peroxidase and SR-A siRNA [108]; SR-A siRNA silences the expression of the SR-A receptor of macrophages, increasing the expression of the CD36 receptor by positive feedback regulation and further increasing the uptake of nanoparticles by macrophages. After 4 weeks of administration, the positive feedback of nanoparticles in the atherosclerotic plaque increases the accumulation by 3.3 times; after 3 months of administration, the plaque area decreases by 65.8%, and the macrophage population decreases by 57.3% [108]. Similarly, CD36 antibody-modified mPEG-PAsp nanoparticles could deliver anti-PAK1 siRNA to atherosclerotic macrophages [109]. CD36 antibody increases the endocytosis of nanoparticles by macrophages, and anti-PAK1 siRNA can negatively inhibit the expression of CD36, thereby reducing the uptake of oxLDL by macrophages and inhibiting the production of foam cells [109].

CD44 is a cell surface receptor overexpressed on cells in atherosclerotic plaques and plays an essential role in plaque formation. The CD44 protein is highly expressed in the macrophages of patients with atherosclerosis [110], implying that CD44 can be used as a target of atherosclerosis macrophages. Hyaluronic acid (HA) is the natural ligand of the CD44 receptor. Iron oxide-based magnetic nanoparticles coated with HA target-activated macrophages for macrophage imaging [111]. LOX-1-specific siRNA nanocomposites coated with HA improve the inherent limitations of siRNA, such as negative charge, considerable molecular weight, instability and target siRNA delivery to macrophages [112]. LOX-1 siRNA can reduce the uptake of LDL by macrophages, thereby inhibiting the progression of atherosclerosis [112]. Atorvastatin is a crucial lipid-lowering agent, but its application is limited due to its hydrophobicity. A new HA-atorvastatin (ATV) conjugate can shield the drug’s hydrophobicity and target atherosclerotic macrophages [113]. *In vitro* studies have shown HA-ATV conjugates have an excellent binding ability with macrophages [113]. After one week of administration, HA-ATV-NPs significantly reduced inflammation in advanced atherosclerotic plaques [113]. Recently, a mechanically sensitive macrophage-targeted HA hydrogel carrier delivers simvastatin to treat atherosclerosis [114]; this new platform can be deformed and reassembled in a 75% occluded simulated blood vessel to produce smaller micelles to target macrophages [114]. Like the CD44 receptor, the scavenger receptor Stabiliin-2 (Stab2) is also considered the main clearance receptor for HA [115]. DSPE-PEG liposomes loaded with CaMKIIγ siRNA and modified with P2P polypeptide target macrophage Stab2 and can silence diseased macrophages CaMKIIγ and promote endocytosis, increase the thickness of the fibrous cap and inhibit atherosclerotic plaque necrosis [116].

In addition, folic acid-modified dendrimers can selectively target folate receptors in atherosclerotic macrophages for atherosclerosis imaging [117]. Tumor homing peptide LyP-1 (CGNKRTRGCk) modified simian virus 40 (SV40) nanoparticles can selectively deliver the anticoagulant hirulog to atherosclerotic macrophages [118]. Furthermore, almost all macrophages express LAF-1 (lymphocyte function-related antigen 1), which can specifically bind to anti-CD11a IgG [119], and the conjugate of anti-CD11a IgG-LXR agonists demonstrated good macrophage affinity *in vitro*.

In addition to macrophage markers, other molecules upregulated in the atherosclerotic lesion can also be used for targeted delivery. For instance, we developed a novel integrin αvβ3 targeted and cathepsin K-responsive nanoparticle (T/R NPs) to control the release of rapamycin in atherosclerosis lesions [120]. The RGD short peptides guide the nanoparticle binding to integrin and cross the endothelial barrier. The cathepsin K sensitive peptide is responsible for the drug releases in response to cathepsin K enzymes enriched in plaques. *In vitro* studies showed that the T/R NPs inhibited the phagocytosis of oxLDL and the release of cytokines by inflammatory macrophages. *In vivo* study demonstrated RAP@T/R NPs significantly blocked the development of atherosclerosis and suppressed the systemic and local inflammation in ApoE^−/−^ mice. In addition, some other drug delivery systems have also been developed to deliver different drugs to atherosclerosis based on targeted scavenger receptors [121–123].

Although targeted drug delivery systems based on receptor-ligand binding have achieved good results in preclinical studies, there are few reports of clinical translational applications. Potential barriers include: (i) the ligand binding is based on weak intermolecular van der Waals force, which is challenging to ensure adequate delivery efficiency. (ii) Most receptors are widely expressed in disease sites and normal tissues, which may lead to off-target side effects of the delivery system.

## Using macrophage membrane-coated carrier for targeted delivery

To achieve more robust targeting, minimize the nonspecific side effects, and avoid fast clearance of drug carriers from the blood circulation, live macrophages have been explored to deliver payloads as they are recruited to atherosclerotic lesions [124]. Drugs encapsulated particles can be loaded in macrophages or on the macrophage surface. However, these approaches are limited by drug loading capacity, drug cytotoxicity, drug stability and the lack of control for release.

One solution is utilizing the biomimetic camouflaging of macrophage membrane-coated carriers, which can protect the drug carrier from clearance by immune cells [125, 126]. In addition, the macrophage membrane retains a part of the targeting capability for atherosclerotic lesions [127], although the chemotactic responses to cytokines in live macrophages are lost. In the past decade, macrophage membrane-coated carriers have been demonstrated as effective delivery systems for atherosclerosis management due to their ability to achieve long-term retention, improved targeting relative to traditional nanoparticles, and low immunogenicity [128–130].

**Figure 5.**
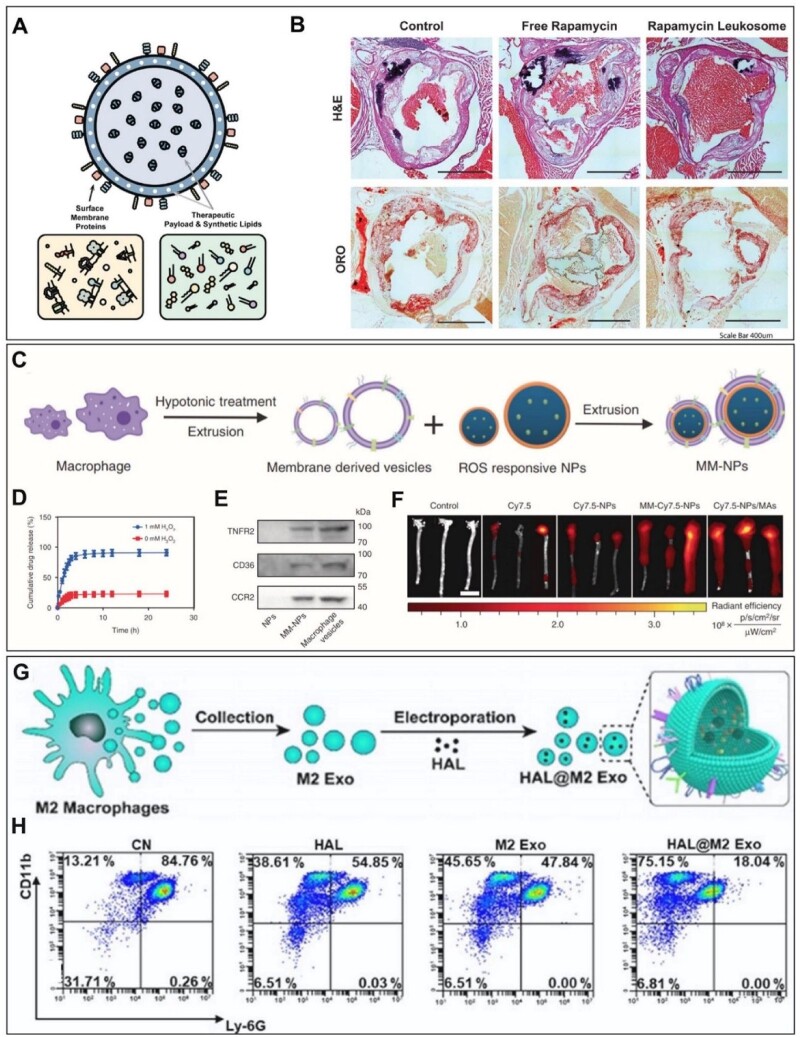
Drug delivery strategies based on macrophage-based cell membrane components or bioactive bodies. (**A**) Schematic representation of leukosome platform [131]. (**B**) RAP-L decreases the plaque burden of atherosclerosis in mice model [131]. (**C**) Schematic illustration of the preparation of MM-at-NPs [133]. (**D**) Drug releases from nanoparticles in different concentrations of H_2_O_2_ [133]. (**E**) Western blot analysis the presence of membrane antigens on the surfaces of both macrophage membrane and MM-NPs [133]. (**F**) *Ex vivo* imaging of Cy7.5 fluorescent signal of MM-Cy7.5-NPs, Cy7.5-NPs/MAs in aorta tissues [133]. (**G**) Schematic illustration of atherosclerosis management of HAL@M2 Exo [135]. (**H**) Flow cytometric analysis of neutrophils in acute peritoneal exudates 24 h after different treatments [135].

Leukosome is a biomimetic vesicle constituted by proteins derived from the leukocytes plasmalemma integrated into a synthetic phospholipid bilayer [131]. The leukosomes retained critical surface proteins such as LFA-1, Mac-1, PSGL-1, CD18, CD45 and CD47, and accumulated in inflamed vasculature due to their ability to extravasate across the vascular barrier. Rapamycin-loaded leukosome can suppress macrophage proliferation within the aorta and potentially stabilize late-stage plaques (Fig. 5A and B).

Alternatively, macrophage-coated nanoparticles, with fewer fabrication steps, possess similar targeting abilities and sequester pro-inflammatory cytokines [132]. Nanoparticles coated in the J774 macrophage cell membrane (MΦ-NPs) act as a decoy to bind cytokines, inhibiting their ability to potentiate downstream inflammation in a concentration-dependent manner due to the macrophage membrane. The MΦ-NPs surface preserved key cytokine binding receptors such as CD126 and CD130 for IL-6, CD120a and CD120b for TNF, and CD119 for IFN-γ.

To achieve tunable delivery, macrophage membrane-coated ROS-responsive nanoparticles are developed to release payload at the diseased site [133]. In this case, atorvastatin-loaded ROS-NP (AT-NP) and macrophage membrane-coated AT-NPs (MM-AT-NPs) is constructed (Fig. 5C). The accumulative drug release rate of AT-NPs increases by ∼80% in the presence of a 1.00 mM H_2_O_2_ environment, whereas only ∼20% of the drug is released without peroxide treatment (Fig. 5D). Additionally, MM-AT-NPs contain key membrane antigens on the surface (e.g. TNFR2, CD36, CCR2), which may sequester pro-inflammatory cytokines or chemokines (Fig. 5E). Interestingly, this study also incubates the AT-NPs with live macrophages to prepare AT-NP/MAs and compared them with the MM-AT-NPs. The NP/MAs exhibited a higher accumulation rate in aorta tissue than MM-NPs (Fig. 5F) due to the stronger targeting capability of live macrophages. However, live macrophages may contribute negatively to the therapy as they are easily activated by inflammatory cytokines or chemokines to release more inflammatory cytokines. In contrast, the macrophage membrane may effectively sequester key pro-inflammatory cytokines or chemokines to decrease their levels in the environment, thereby reducing local inflammation.

To circumvent the expensive membrane collection associated with membrane-coating nanoparticles, researchers have also used exosomes, extracellular vesicles containing the parent cells’ constituents [134]. For example, molecularly engineered M2 macrophage exosomes (M2 Exo) have inherent inflammation tropism and intrinsic bifunctionally targeting (Fig. 5G) [135]. M2 Exo is electroporated with FDA-approved hexyl 5-aminolevulinate HAL (HAL). The HAL@M2 Exo could significantly decrease the production of ROS and iNOS in inflammatory cells compared with HAL or M2 Exo alone. The HAL@M2 Exo was also found to induce the phenotype change of M1 cells by increasing M2 biomarkers, CD206 and CD163, and decreasing M1 biomarkers, CD80 and CD86 (Fig. 5H). *In vivo* studies showed reduced inflammation-induced aorta lesion area and aortic valve lesion in groups treated with HAL@M2 Exo. It is worth noting that exosomes may contain a mixture of components, and batch-to-batch variations need to be addressed.

## Conclusion and future directions

Macrophages play an essential role in the development of atherosclerosis. The lipid uptake and phenotype of macrophages are affected by their microenvironment and perform different functions. Ideally, macrophages should uptake and metabolize lipids deposited on blood vessel walls, reduce the production of pro-inflammatory phenotype macrophages, and reduce the release of inflammatory factors, but the formation of foam cells and chronic inflammatory responses in the lesion areas cause the disease development. Therefore, understanding and controlling macrophage phenotype and functions in atherosclerosis is critical for therapeutic development.

Further analysis at the single-cell level is needed to gain insight into the macrophage phenotypes and the interactions of macrophages with other immune cells and vascular cells. Recently, single-cell sequencing of immune cells in human aortic plaques reveals that the predominant immune cell types are macrophages and T cells [136], and the proportion of macrophages in the plaque is about 16%. Further analysis of macrophages in plaques revealed more significant functional heterogeneity, including clusters with activated and pro-inflammatory phenotypes and a cluster with a foam cell transcriptional signature. Single-cell sequencing, together with spatial transcriptome analysis, can provide cellular profiles with higher temporal and spatial resolutions. Temporal studies can elucidate the dynamic changes of macrophage phenotype, rather than static or simple maintenance of a particular phenotype. In addition, the single-cell analysis will also allow us to better dissect cell–cell interactions in the lesion area. All of these studies will help us understand new mechanisms and the key factors that regulate the macrophage phenotype and identify potential therapeutic targets.

Besides the discovery of novel therapeutics, the delivery platforms need to be further optimized. Intravenous injection of nanotherapeutics is the most effective route of administration for their systemic infiltration into atherosclerotic plaques. After entering the blood circulation, the proteins in the blood will aggregate around the nanoparticles to form nanoparticle protein corona, which affects physiological response including pharmacokinetics, cellular uptake and biodistribution. Current preclinical studies of atherosclerosis targeting strategies are mainly to prolong the blood circulation time of the carriers by surface modification, such as PEG or biomimetic materials such as macrophage membrane, erythrocyte membrane, platelet membrane and exosomes [124]. After circulating in the blood, efficient accumulation of nanoparticles in diseased macrophages relies on either passive extravasation of leaky vasculature or active targeting combined with endocytosis. For example, nanoparticles modified with macrophage membranes can significantly improve the blood circulation time of nanoparticles and increase aggregation in plaques [133]. How to scale up the production of cell membrane components for nanoparticle coating remain to be addressed. Alternatively, we may take a synthetic biology approach to design and synthesize a liposome-based delivery system. In addition, the size and shape of nanoparticles also significantly influence the biodistribution of nanocarriers. The researchers found that nanoparticles with smaller diameters (7–30 nm) accumulated more in the plaques than those with larger diameters (>70 nm) [137]. Rod-shaped, cylindrical nanocarriers and nanodiscs have greater vascular tissue penetration over spherical nanoparticles [138, 139]. However, the effects of nanoparticle size, shape, charge, chemical composition and surface functional groups and targeting ligands on their distribution in plaques remain largely unexplored and warrant future systematic studies. Finally, genetically engineered immune cell therapies have shown promising results in cancer treatment. There is a potential to engineer macrophages and other immune cells with desirable phenotypes and functions to develop innovative immunotherapy for atherosclerosis.
